# A systematic review of delay discounting among workers: framing effects, resource availability, and health

**DOI:** 10.3389/fpsyg.2026.1801136

**Published:** 2026-07-13

**Authors:** Gisel G. Escobar, Cynthia Zaira Vega Valero

**Affiliations:** Department of Psychology, School of Higher Studies Iztacala, National Autonomous University of Mexico, State of Mexico, Mexico

**Keywords:** delay discounting, framing, methodologies, organizations, workers

## Abstract

A higher rate of delay discounting has been linked to a range of health-risk behaviors and adverse health outcomes. Despite its relevance, delay discounting has limited application in organizational contexts. The aim of this systematic review was to identify empirical studies on the application of delay discounting in organizations or among workers. Sixteen studies were included. Overall, contextual factors (resource availability and negative income shock) and methodological factors (framework, reward magnitude, task type) influenced delay discounting. Consistent effects were observed, including preference reversals, magnitude and sign effects, and clear distinctions between delay and probability discounting. Limitations include insufficient characterization of organizational variables, insufficient information on data systematicity and fit level of the mathematical discounting functions. Despite these limitations, delay discounting proved to be a sensitive and applicable behavioral indicator of increased self-control, capable of predicting adherence to a physical activity intervention in sedentary workers. The review also identifies opportunities for future research, including multidimensional delay discounting analysis, inter-outcome tasks, and the integration of comparison groups in health-related studies. Overall, delay discounting provides a practical framework for studying decision-making in organizational contexts and for guiding interventions to improve workers' self-regulation and long-term goal achievement.

## 1 Introduction

According to the American Psychological Association (American Psychological Association, 2022), organizational or industrial psychology is the discipline devoted to the scientific study of human behavior in organizations, with the aim of extrapolating principles of individual behavior to group behavior in organizational settings to address workplace problems. Within this context, decision-making constitutes one of the most critical organizational processes, as it serves as the starting point for virtually all activities, both within and beyond the work environment (Bahrawi and Ali, 2023). Decision-making occurs across different hierarchical levels of the organization, including both managers and employees. A fundamental characteristic of many organizational decisions is that they involve trade-offs between short- and long-term consequences. For example, pursuing a degree, engaging in physical exercise, changing jobs, making investments, or saving for retirement are actions whose outcomes unfold in both the present and the future. Decisions of this kind are referred to as intertemporal choices, which involve selecting among alternatives that yield consequences at different points in time (Loewenstein, 1987).

In the workplace context, (Kozioł-Nadolna and Beyer 2021) proposed that workers' decisions can be classified into three types according to the temporal horizon of their outcomes. Strategic decisions are oriented toward achieving long-term results, such as defining policies aligned with the organization's vision and mission. Tactical decisions yield medium-term outcomes, for instance, selecting the most suitable individual to lead the organization and subsequently influence strategic decision-making. Finally, operational decisions are made in day-to-day work activities. They are associated with short-term outcomes, such as prioritizing complex tasks over simpler ones to enhance productivity and reduce the accumulation of critical work at the end of the workday.

Decision making involves a sequence linking available alternatives, workers' behavior, the resulting outcomes, and the resolution of the event in question. Decisions become complex because, in some cases, alternatives may differ along more than one dimension—for example, in the magnitude or value of the outcome, as well as in the time or probability required to obtain it. Within organizations, workers are frequently exposed to such decision scenarios, in which they must make trade-offs or evaluative judgments to select the alternative that best serves a particular function in the work environment.

For instance, employees may postpone enjoyable leisure activities (e.g., checking social media) to devote additional time to completing a monthly report. Another example involves wearing personal protective equipment incorrectly to save time, rather than spending a few extra minutes putting it on properly to prevent future accidents. The first example represents a form of self-controlled, operational choice, as the task completion at the end of the workday (a delayed outcome) is prioritized over the immediate gratification of checking social media at work. In contrast, the second example reflects an impulsive, operational choice, as immediacy—quickly putting on safety equipment incorrectly—is prioritized over the delayed consequences of a potential workplace accident. Preference for smaller, immediate outcomes over larger, delayed outcomes is commonly regarded as a departure from rationality in intertemporal choice or as impulsive decision making (Green and Myerson, 2004; Odum, 2011).

There are situations in which individuals tend to make decisions that yield poor and immediate outcomes, even when it is evident that alternative options would provide greater benefits in the future. In behavior analysis, such an apparently irrational choice is examined using the delay discounting paradigm. Delay discounting is the decrease in the subjective value of an outcome (a reward or an aversive event) as a function of the delay to its occurrence (Rachlin et al., 1991). In humans, delay discounting has been studied predominantly using hypothetical monetary rewards. For example, participants may be asked to choose between receiving $500 now or $1,000 in 1 month. Across trials, the amount of the smaller–sooner alternative is adjusted, whereas the larger–later amount remains fixed within each block of delays, which is defined by a specific delay interval (e.g., 1 month, 3 months, 1 year). Based on each block of trials, the researchers calculate the indifference point, defined as the value of the immediate alternative that the participant subjectively considers equivalent to the larger, delayed alternative. At this point, the participant shows no systematic preference for either option.

Typically, participants are instructed to make their choices as if they were going to receive the monetary amounts and experience the specified delays. A substantial body of research has shown that delay discounting follows a hyperbolic function; that is, short delays produce a sharper reduction in the subjective value of an outcome than longer delays (Green and Myerson, 2004; Odum, 2011). This pattern, known as preference reversal, assumes that individuals change their initial preferences, often opting for a smaller, immediate reward over a larger, delayed one, even when the options are objectively identical. Evidence of preference reversal stands in contrast to classical utility theory (Samuelson, 1937) and indicates that choice behavior is context-dependent and sensitive to the decision-making procedure. This discounting function is commonly described mathematically using the equation proposed by Mazur (1987):


$$\begin{array}{l} V=\frac{A}{\left(1+k\;D\right)}, \end{array}$$ (1)

where *V* represents the subjective value of the outcome, *A* denotes the maximum magnitude of the outcome, *D* corresponds to the delays employed, and *k* is a free parameter that reflects the rate of temporal discounting. The model assumes that *V* decreases sharply in response to short delays.

Delay discounting is recognized as a state-like variable with a certain degree of temporal stability (Odum, 2011). In this regard, delay discounting has gained prominence in behavioral psychology and related disciplines for at least three reasons. First, a wide range of experimental contexts can be used to design discounting tasks, and these contexts systematically influence impulsive choice. For example, the magnitude effect indicates that larger delayed rewards are discounted less than smaller immediate rewards (Kirby, 1997); the sign effect for gains and losses shows that gains are discounted more steeply than losses (Green et al., 2014); and the domain effect (Odum and Rainaud, 2003) demonstrates that consumable rewards (e.g., food) are discounted more steeply than non-consumable rewards such as money. Second, numerous studies have shown that individuals with substances use and abuse (Bickel et al., 1999; MacKillop et al., 2010; Mejía-Cruz et al., 2016), obesity (Lawyer et al., 2015), those who engage in risky sexual behaviors (Sweeney et al., 2020), and those with lower treatment adherence (Campbell et al., 2021), among other health-related problems, consistently exhibit higher rates of delay discounting (i.e., greater impulsivity) than individuals without such conditions. Consequently, delay discounting has been conceptualized as a transdiagnostic process (Amlung et al., 2017; Bickel et al., 2012). The third reason is closely related to the second: the Area Under the Curve ([AUC]; Myerson et al., 2001) and/or the discounting rate are frequently used as primary or complementary indicators of behavioral change following an intervention. That is, participants typically exhibit reduced delay discounting (i.e., greater self-control) following effective interventions (Rung and Madden, 2018).

Reynolds and Schiffbauer (2004) published one of the earliest articles calling for applying principles from the discounting paradigm to the field of occupational safety and health, through a multidimensional approach in which different choice costs—such as delay, probability, and effort requirements—converge simultaneously to yield outcomes. Reynolds and Schiffbauer (2004) emphasized that, in real workplace settings, safety-related decisions (e.g., wearing complete protective equipment to prevent accidents) often involve multiple forms of discounting operating simultaneously. For example, when deciding whether to use personal protective equipment, workers may prefer the immediate benefits of time savings and comfort over the unlikely but severe future cost of an accident.

Nevertheless, there seems to be limited empirical evidence examining delay discounting specifically among workers. For instance, Hesketh (2000) assessed choice behavior in expert career counselors vs. a group of university students with no work experience, using two discounting tasks that combined delays to employment outcomes and the degree of match between academic training and job type. These tasks were applied to both personal decisions and third-party decisions involving recent graduates. The results showed that experts, compared to novices, judged that graduates would be more willing to accept immediate but lower-quality jobs rather than wait for better employment opportunities, particularly at short delays (1–3 months). This finding suggests that experts perceive graduates as less “wise” or less self-controlled in their career-related decisions. Similarly, Logue and Anderson (2001) found that provosts and experienced fellows discounted organizational financial resources more steeply than new fellows. This finding suggests that organizational context—particularly perceptions of resource availability—plays a critical role in shaping workers' behavior and their decisions regarding resource management.

Both the proposal by Reynolds and Schiffbauer (2004) and the seemingly limited empirical evidence on delay discounting among workers, encourages further investigation into whether the principles derived from discounting models extend to workplace contexts. If so, such findings would support generalizing these models beyond laboratory settings. If not, identifying key parameters—such as the magnitude, delay, and frequency of outcomes—in the choices of employed individuals could nonetheless improve working conditions. Therefore, this systematic review aimed to identify empirical studies on the application of the delay discounting paradigm in organizations or among workers, and to examine the methodologies used, the contexts, and the types of work samples studied.

## 2 Methods

The present systematic review was conducted in accordance with the guidelines of the Preferred Reporting Items for Systematic Reviews and Meta-Analyses (PRISMA; Moher et al., 2009; Page et al., 2021). Based on the stated aim, two research questions arose: What methodologies and metrics are used to assess delay discounting in worker samples? And in what types of organizational contexts and work samples has delay discounting been studied, and what are the main findings reported?

### 2.1 Eligibility criteria

We aimed to include studies that applied methods to measure delay discounting in samples of workers. The inclusion criteria were as follows: (i) empirical articles that estimated discounting metrics (e.g., discount rates or*k*-values, area under the curve, indifference points, proportion of choices, or other analogous measures) in worker samples; (ii) articles published with no start-date restriction up to June 2025; (iii) articles published in Spanish or English; and (iv) peer-reviewed articles.

Articles were excluded if they represented: (i) thesis, theoretical papers, conference abstracts, unpublished data, preprints, or similar sources; (ii) literature reviews; (iii) if they relied on pre-existing data to estimate delay discounting rates; (iv) if they did not provide a clear and sufficiently detailed description of the discounting task employed, such that the nature of the task, the manipulated variables, or the dependent variable used for data analysis was unclear; (v) if the delay discounting task was designed for a workplace context but the sample consisted of students; or (vi) the sample was not explicitly described as comprising workers of any kind (e.g., teachers, administrative staff, waitstaff, call center operators, among others).

### 2.2 Information sources and search strategy

Three databases were used for the literature search conducted in June 2025. First, PsycInfo was selected because it covers a broad range of psychological literature, along with two multidisciplinary databases: the Wiley Online Library and Web of Science. Using the advanced search options of each database, two search strings were defined and applied simultaneously using the Boolean operators OR and AND to search titles, abstracts, and keywords.

The first search string included the following terms: “delay discounting” OR “time discount” OR “temporal discount” OR “intertemporal choice” OR “inter-temporal choice” OR “time-related discount” OR “impulsive choice” OR “choice impulsivity” OR “delay discount.” Subsequently, the operator AND was applied to a second search string related to the target sample: “employee” OR “organization” OR “workplace” OR “work” OR “career” OR “organizational” OR “job.” Some terms from the first search string were adapted from the systematic review by Lipman and Attema (2024).

The search strategy employed for the present review (conducted by the first author) yielded 423, 64, and 288 records in PsycInfo, Wiley Online Library, and Web of Science, respectively. A common characteristic of journals that publish articles in Spanish is that authors are required to provide English versions of the title, abstract, and keywords, even when the remainder of the manuscript is in Spanish (e.g., *Revista Mexicana de Análisis de la Conducta, Conductual, Acta Comportamentaliay*). For this reason, Spanish-translated search terms were not used when searching for articles published in Spanish.

### 2.3 Screening

**Figure 1:**
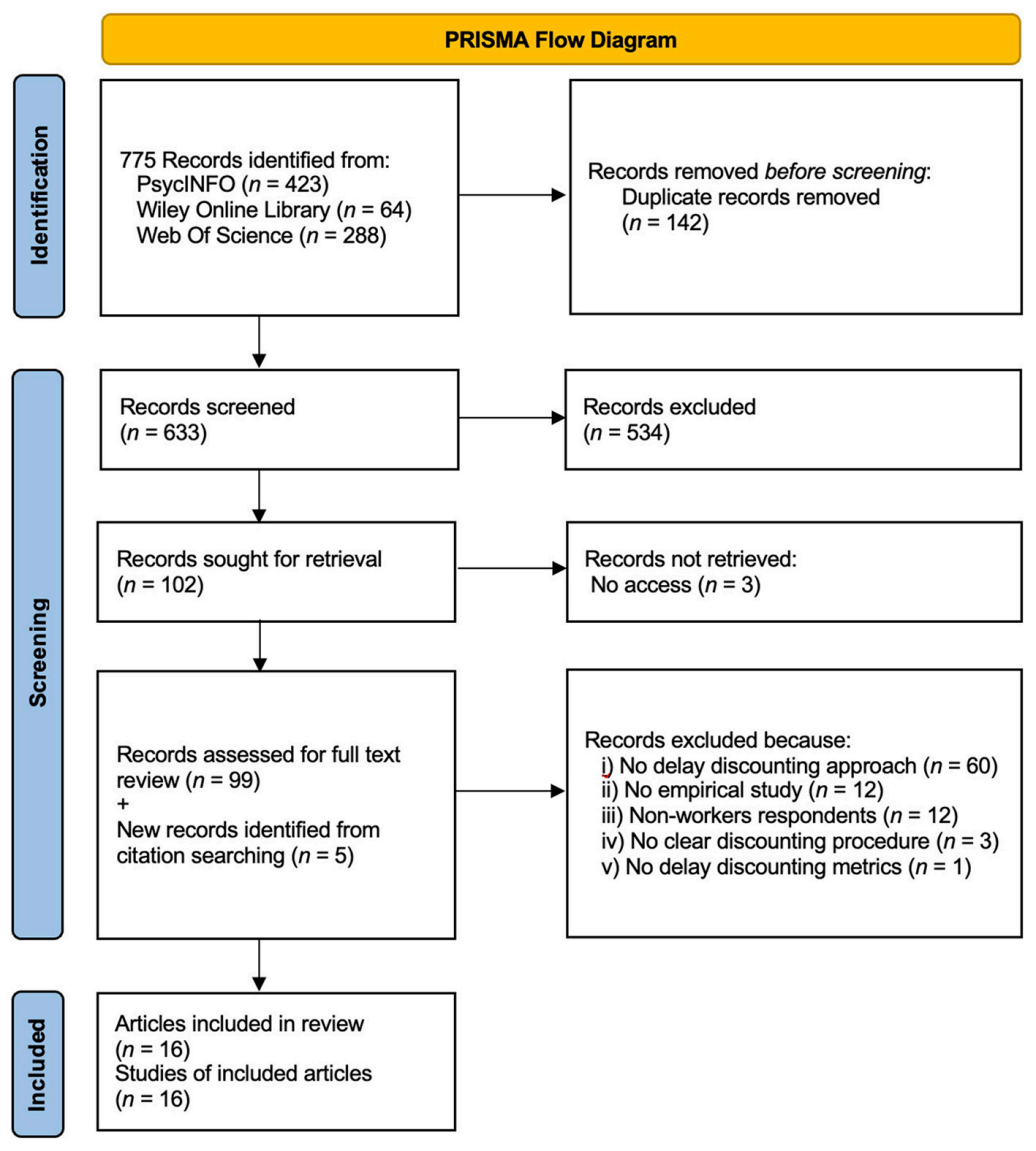
Preferred reporting items for systematic review and meta-analyses (PRISMA) flow diagram. Although some articles contained more than one study, 16 studies met the inclusion criteria for the review.

The first author used the Rayyan.ai tool to identify and remove duplicate records (see Figure 1 for the systematic review flow diagram). Subsequently, Microsoft Excel was used to screen the titles, abstracts, and keywords of the 633 records to identify at least two terms drawn from both search strings. For example, if only one term from one search string was identified and no term from the second search string was found, the article was excluded. In contrast, if one term from one search string appeared in the title and a second term from the other search string was identified in the abstract or keywords, the article was retained for full-text review.

Following this initial screening by the authors, 102 records remained, of which three additional articles were excluded because they could not be retrieved via the databases accessible at our university or were not available via open access. Ninety-nine articles were then screened in full text by both authors to verify compliance with the inclusion criteria or to exclude articles based on the exclusion criteria. This process also led to the identification of five additional articles through reference lists of the screened studies, which were evaluated using the same criteria to determine their eligibility. The final screening yielded 16 articles that met the inclusion criteria and were included in the qualitative analysis of the systematic review (Figure 1).

### 2.4 Data extraction

The authors independently reviewed each study and reached consensus on the categories used to organize the methodological characteristics of the included studies, with emphasis on the type of workers sampled, study design, delay discounting procedures, data analysis strategies, and main findings. When there was any doubt regarding the classification of information from an article, the researchers reviewed it together to decide on its proper inclusion. A narrative synthesis approach was used to summarize and interpret the study findings (Popay et al., 2006). This approach comprises four key elements: (i) employing a theoretical framework to ground the analysis of findings and their implications for knowledge generation—in this case, the delay discounting as a behavioral approach to the study of impulsive choice in organizational contexts; (ii) developing a preliminary synthesis that provides an overall mapping of findings across the selected studies; (iii) exploring relationships among study methodologies and findings through information groupings constructed by the authors; and (iv) assessing the robustness of the narrative synthesis and the quality of the evidence reported in the included studies.

## 3 Results

The flow diagram (Figure 1) illustrates the search strategy, which yielded 16 studies that met the inclusion criteria for the present systematic review. The following sections describe the risk-of-bias assessment, followed by an overview of the general characteristics of the included studies, first detailing their methodological aspects and subsequently grouping the evidence into selected thematic topics.

### 3.1 Risk of bias

The authors assessed the risk of bias for each included article using tools developed by the National Institutes of Health (NIH; National Heart, Lung, and Blood Institute, 2019): the *Quality Assessment Tool for Observational Cohort and Cross-Sectional Studies*, comprising 14 criteria, was applied to 14 studies; and the *Quality Assessment Tool for Before–After (Pre–Post) Studies With No Control Group*, comprising 12 criteria, was applied to one study (Losina et al., 2017). The study by Saunders and Fogarty (2001) could not be fully evaluated using the NIH tools because of its longitudinal design, for which no specific NIH assessment instrument is available. However, eight criteria from the Quality *Assessment Tool for Observational Cohort and Cross-Sectional Studies* applied to the study by Saunders and Fogarty (2001), resulting in an overall rating of “fair.” Overall, this phase corresponds to the fourth element of the narrative synthesis. Fifteen studies evaluated using NIH tools received ratings of “fair” or “good.” It is worth noting that 6/14 criteria included in the Quality *Assessment Tool for Observational Cohort and Cross-Sectional Studies* did not apply to the reviewed studies, as they are specific to cohort designs that involve exposure to risk factors vs. non-exposure. Consequently, none of the 14 studies assessed with this tool could attain the maximum “good” rating (see Supplementary Tables 1 and 2 for detailed results).

Additionally, several NIH criteria overlapped with the a priori inclusion criteria established for this review, such as precise descriptions of manipulated variables, dependent variables, and worker-based samples. Taken together, this overlap and the predominance of “fair” and “good” ratings suggest that the inclusion criteria proposed for this review are consistent with established standards for selecting studies of acceptable methodological quality.

### 3.2 General characteristics of selected studies

The literature search yielded 16 articles published between 2000 and 2025, distributed across 11 scientific journals: *Appetite* (*n* = 2), *BioMed Central Public Health* (*n* = 1), *Frontiers in Psychology* (*n* = 1), *Judgment and Decision Making* (*n* = 1), *Journal of Counseling Psychology* (*n* = 1), *Journal of Organizational Behavior Management* (*n* = 2), *Journal of Vocational Behavior* (*n* = 3), *PLoS ONE* (*n* = 1), *Psychological Science* (*n* = 2), *Public Management Review* (*n* = 1), and *The Journal of Socio-Economics* (*n* = 1). Of these 11 journals, two can be considered specialized in organizational research: *Journal of Organizational Behavior Management* and *Journal of Vocational Behavior*. The remaining studies were published in journals focused on general psychology, multidisciplinary research, or health-related topics.

The Americas account for the most significant proportion of included studies (56.25%), driven primarily by the United States (50.0%, *n* = 8) and Mexico (6.25%, *n* = 1). This is followed by the Asia–Pacific region, which collectively represents 31.25% of the studies, including China (6.25%, *n* = 1), Hong Kong (6.25%, *n* = 1), Israel (12.5%, *n* = 2), and Australia (12.5%, *n* = 2). Finally, Europe contributes 6.25% (*n* = 1), with Germany as the sole contributor (Table 1). All included studies were published in English.

**Table 1:** General characteristics of the 16 included studies.

References	Country	Design	Sector	Sample type	Manipulation	Outcome type	# Delays
Bickel et al. (2016)	USA	E	Private	AMT	Framing; Sign; Past-Future	Money	7
Bidewell et al. (2006)	Australia	Q	Public/Private	Educational staff	Framing	Money	2
Dixon et al. (2018)	USA	F	Private	Waitresses	Titration	Money	7
Duan et al. (2017)-ex2	China	Q	Private	Corporate employees	Framing	Air quality	1
Hernández-Toledano et al. (2025)	Mexico	Q	Private	Managers & operative workers	Framing; Amount	Money	7
Hesketh (2000)-ex2	Australia	Q	Private	Career advisers and counselors	Framing	Job fit	7
Joshi and Fast (2013)-ex1	USA	E	Private	AMT	Framing	Money	1
Lahav et al. (2011)	Israel	F	Army	Soldiers	Framing; Amount	Money	3
Logue and Anderson (2001)	USA	Q	Private	Provost, experienced fellows, & new fellows	Framing	Money	8
Losina et al. (2017)	USA	Q	Public/Private	Non-clinician employees	Intervention	Money	9
Mellis et al. (2018a)	USA	E	Private	AMT	Framing; Cross-commodity	Money/Food gift card	7
Mellis et al. (2018b)	USA	E	Private	AMT	Framing	Money	7
Saunders and Fogarty (2001)	Hong Kong	L	Private	High staff members	Framing	Job promotion	4
Shavit et al. (2013)	Israel	F	Army	Soldiers	Framing; Amount	Money	3
Weißmüller (2022)	Germany	E	Public/Private	General workers	Framing; Amount	Money	9
Xu and Yin (2020)	USA	Q	Private	AMT	Framing	Money	7

E, experimental design; Q, quasiexperimental; L, longitudinal; F, field study; AMT, Amazon Mechanical Turk workers.

Three articles reported more than one study (experiment) within the same publication. However, only those studies that included worker samples were retained for this review (*n* = 3), and six studies from these articles were excluded (Duan et al., 2017; Hesketh, 2000; Joshi and Fast, 2013). In the tables presented in this review and in the description of results, reference is made to the specific experiment number included (-exp#) from each of the articles.

#### 3.2.1 Worker's characteristics

The present analysis of the 16 included studies comprises a total of 2,826 workers from the private sector (69%), the mixed public–private sector (19%), and the army (13%). Sample sizes ranged from 13 to 595 workers (*M* = 128.45; *SD* = 154.56). There was substantial heterogeneity in occupational type, with 31% of the samples representing a diverse range of occupations, including administrative staff, teachers, professional counselors, exotic dancers, waitstaff, hospital employees, and security guards (see Table 1). These were followed by samples of Amazon Mechanical Turk (MTurk) workers (31%), managers or provosts (25%), and soldiers (13%). A single study relied exclusively on female samples (Dixon et al., 2018); the remaining studies included workers of both biological sexes (women and men). The age of workers ranged from 18 to 65 years. Because not all studies reported means and/or standard deviations for participant age, only the overall age range is reported. Similarly, relatively few studies reported years of work experience; among those that did, the mean was 6.95 years (a standard deviation could not be calculated due to missing data in some studies).

#### 3.2.2 Outcomes and delay characteristics

The following description includes the delays in the selected studies, which were converted to days for descriptive purposes in this review. The range of delays used per study was 1 to 9 (*M* = 5.56; *SD* = 2.75). The range of delays converted to days was 0.041 to 9126, with the shortest delay being 1 day and the longest 25 years. The studies differed considerably in the breadth of time horizons evaluated and the number of delay intervals used, demonstrating significant methodological heterogeneity across the delay discounting tasks employed.

Twelve studies used monetary rewards, and one study used both monetary rewards and food vouchers. The remaining studies (*n* = 3) assessed occupationally relevant or work-context-specific non-monetary outcomes, such as air quality, job-career fit, and career advancement (Table 1). The maximum amounts used depended on the type of outcome and the number of magnitudes. All studies included hypothetical rewards.

Although most studies used hypothetical monetary rewards, the diversity of non-monetary outcomes is relevant, as delay discounting rates are known to differ depending on the type of reward (consumable vs. non-consumable; see the meta-analysis by Odum et al., 2020), the magnitude of the outcome, and its contextual relevance. Compared to monetary rewards, domain-specific, non-monetary outcomes may involve different choice processes, which could limit direct comparability between studies. For example, only the study by Mellis et al. (2018a) compared monetary rewards and credit for food purchases in two framing scenarios (negative income shock and neutral). The results showed higher delay discounting rates (k-values) in the negative framing scenario, particularly for fast food, compared with the neutral framing scenario for bottled water, where delay discounting was lower. That is, in situations of uncertainty and financial scarcity, immediate monetary rewards, especially those from fast food, are prioritized.

The other three studies that used non-monetary outcomes (Duan et al., 2017-ex2; Hesketh, 2000-ex2; Saunders and Fogarty, 2001), although they were able to increase the ecological validity of their findings by using rewards contextualized to decisions according to the type of sample and work scenario, also have limitations on the standardization of their procedures, making comparisons with other delay discounting studies difficult.

#### 3.2.3 Study design and discounting procedures

Only the study by (Saunders and Fogarty 2001; 6%) was longitudinal (with measurements at baseline and at 13, 25, and 36 months of follow-up) in a sample of employees participating in a career advancement training program. Lahav et al. (2011), Shavit et al. (2013), and Dixon et al. (2018) conducted field studies (19%, *n* = 3). Thirty-one percent (*n* = 5) of the studies used an experimental design, while the remainder employed a quasi-experimental design (*n* = 7). The heterogeneous methodology of the designs used also has implications for the conclusions regarding the effect of experimental arrangements on delay discounting.

Two studies used discounting tasks combining delays and percentages related to health and retirement enjoyment (Bidewell et al., 2006), as well as delays and percentages related to job-career fit (Hesketh, 2000-ex2). Although these studies combined delays and the percentages of a given outcome within the same trial, they did not assess probability discounting: the loss of the subjective value of an outcome as a function of the odds against its occurrence (Rachlin et al., 1991). In contrast, the study by Weißmüller (2022) assessed both delay and probability discounting using independent tasks. The remaining studies only assessed temporal discounting.

Similarly, heterogeneity was observed in the strategies used to calculate indifference points or discount rates (k-values). We identified seven different procedures: Monetary Choice Questionnaire (Kirby et al., 1999; *n* = 2), adjusting-amount procedure (Du et al., 2002; *n* = 2), 5-trial adjusting delay procedure (Koffarnus and Bickel, 2014; *n* = 2), titration—fixed alternative survey—procedure ascending and descending orders (Rachlin et al., 1991; *n* = 4), titration procedure with lottery commission (Hardisty and Weber, 2009; *n* = 2); procedure to calculate the subjective annual discount rate (Equation 2; *n* = 2); and two studies that combined procedures for calculating delay discount with non-monetary outcomes (Hesketh et al., 1998; Rachlin et al., 1991). Within the included studies, 1 to 4 delay discounting tasks were administered (*M* = 2; *SD* = 1), mostly completed by the same participants, except in one study (Mellis et al., 2018b). In studies that presented more than one discounting task, experimenters counterbalanced or randomized the tasks to reduce order effects. On average, 92.31 (*SD* = 125.16; range 6–472) trials were used per study. Most studies presented the discounting tasks using a computer (*n* = 13). In contrast, one study used both computer and paper-and-pencil tasks (Dixon et al., 2018), and two studies used paper-and-pencil tasks, due to the quasi-experimental and field design (Lahav et al., 2011; Shavit et al., 2013).

Of the different experimental arrangements of the discounting area, 5 studies in this review evaluated the magnitude and framing effect, 1 study jointly evaluated framing, sign effect, and past and future choices (Bickel et al., 2016), and only one study measured delay discounting in combination with framing and cross-commodities (Mellis et al., 2018a). The remaining studies used different narratives to evaluate their effects on delay discounting.

#### 3.2.4 Dependent variables and data analysis

Table 2 summarizes the characteristics of the metrics used in the 16 included studies, along with the mathematical models applied. The most frequently used metric was the *k*-values (9/16 studies) derived from the hyperbolic model. Other metrics included the *area under the curve* (AUC; 3/16 studies), calculated using either the method proposed by Myerson et al. (2001), the method introduced by Killeen (2023), or an area-over-the-curve transformation; *indifference points* (2/16 studies); the *annual subjective discount rate* (2/16 studies); and three additional approaches used to compute analogs of indifference points (3/16 studies): the *switch point* (Saunders and Fogarty, 2001), the *average amount of money sacrificed for early retirement* (Bidewell et al., 2006), and the *average percentage match point within each time delay* (Hesketh, 2000, Exp. 2). Few studies employed combinations of the metrics described above.

**Table 2:** Mathematical modeling characteristics of the 16 included studies.

References	Dependent variable	Mathematical models	Level of analysis
Bickel et al. (2016)	*k*-values	Equation 1	Aggregate
Bidewell et al. (2006)	Average amount of money sacrificed for early retirement	NA	Aggregate
Dixon et al. (2018)	IP & AUC	Equations 1, 2	Individual & Aggregate
Duan et al. (2017)-ex2	*k*-values	Equation 1	Aggregate
Hernández-Toledano et al. (2025)	*k*-values & AUC^*^	Equation 1	Aggregate
Hesketh (2000)-ex2	*k*-values & Average of percentage match point by delay	Equations 1, 3	Aggregate
Joshi and Fast (2013)-ex1	*k*-values	Equation 1	Aggregate
Lahav et al. (2011)	Annual subjective discount rate	NA	Aggregate
Logue and Anderson (2001)	*k*-values & IP	Equation 1	Aggregate
Losina et al. (2017)	*k*-values	Equation 1	Aggregate
Mellis et al. (2018a)	*k*-values	Equation 1	Aggregate
Mellis et al. (2018b)	*k*-values	Equation 1	Aggregate
Saunders and Fogarty (2001)	Point of change	NA	Individual & Aggregate
Shavit et al. (2013)	Annual subjective discount rate	NA	Aggregate
Weißmüller (2022)	*k*-values & *h*-values	Equation 1	Aggregate
Xu and Yin (2020)	IP & Area above the curve	NA	Aggregate

Equation 1: Hyperbolic (Mazur, 1987). Equation 2: Hyperboloid (Myerson and Green, 1995). Equation 3 Exponential (Samuelson, 1937). NA, no applicable; IP, indifference points; AUC, area under the curve. Studies in bold reported fit indicators for the mathematical models used. ^*^AUC calculated with Killeen's method 2023.

Most studies relied on descriptive and inferential statistics to examine associations between delay discounting and demographic variables. Four studies applied advanced statistical techniques—such as multilevel mixed-effects models, structural equation modeling, and generalized estimating equations—to handle hierarchical data structures and to model complex relationships between delay discounting and latent constructs (e.g., satisfaction, optimism, delay gratification, sense of power).

Although the criteria proposed by Johnson and Bickel (2008) are widely regarded as vital benchmarks for assessing data validity in discounting research, only two studies have applied them (Hernández-Toledano et al., 2025; Mellis et al., 2018b). The study by Dixon et al. (2018) implemented the criteria described by Dixon et al. (2003) to identify anomalous response patterns and exclude non-monotonically decreasing data. Taken together, these findings indicate limited evidence regarding the level of data systematicity in delay discounting measures obtained from worker samples.

The most frequently used discounting model was the hyperbolic function (Equation 1), which was employed in 8 studies. This was followed by the hyperboloid model (Equation 2; Myerson and Green, 1995) and the exponential model (Equation 3; Samuelson, 1937), each reported in a single study. Two studies explicitly compared alternative mathematical functions: hyperbolic vs. hyperboloid (Dixon et al., 2018) and hyperbolic vs. exponential (Hesketh, 2000, Exp. 2). In these comparisons, the best-fitting model varied by outcome combination, with the hyperboloid model showing superior fit in Dixon et al. (2018) (*R*^2^ ≥ 0.98) and the hyperbolic model outperforming the exponential function in Hesketh (smaller mean square error). Notably, the remaining six studies that applied the hyperbolic model did not report goodness-of-fit indices (e.g., neither *R*^2^ nor mean square error). Overall, 88% of the studies reported only group-level analyses, whereas the remaining studies presented both individual- and group-level data. The hyperboloid model represents an extension of the hyperbolic function and is defined as:


$$\begin{array}{l} V=\;\frac{A}{\left(1+kD^{s}\right)} \end{array}$$ (2)

where the parameters are the same as in Equation 1, and *s* represents a second free parameter that indexes the scaling of delay and amount. The hyperboloid model does not assume preference reversals; however, the inclusion of the additional free parameter allows for greater precision when fitting human data in cases where the discount rate is not constant (Myerson and Green, 1995). The exponential model is described as Samuelson (1937):


$$\begin{array}{l} V=Ae^{-k\;D} \end{array}$$ (3)

where the parameters are the same as in Equation 1, while *e* represents the exponent, the exponential model also does not assume preference reversals, as it posits that the discount rate remains constant over time (rational choice).

### 3.3 Evidence grouped by topics

The qualitative analysis of the findings was conducted and presented in an integrated manner, organized around selected topics within the delay discounting and organizational domains.

#### 3.3.1 Framing

According to Tversky and Kahneman (1981), framing, or decision framing, refers to the context in which a decision is made, including the behaviors and contingencies associated with a particular choice. Framing can involve norms, habits, the decision-maker's individual characteristics, or different versions of the same situation. In the same work, the authors extend the concept of framing to the way a choice is presented in terms of monetary gains or losses, a phenomenon known as the sign effect or gain-loss asymmetry (Baker et al., 2003), in which outcomes that constitute a gain are discounted at a higher rate than outcomes that constitute a loss.

For clarity purposes in this review, we will distinguish between the meaning of framing and the sign effect. By framing, we refer to broader contextual or narrative framing of the decision scenario, while the sign effect, as mentioned in Table 1, refers to the asymmetric effect of delay discounting on gains and losses.

Analysis of the articles selected for this review revealed that the majority (14 of 16 studies) employed narratives or hypothetical decision scenarios that fit the previously described definition of framing (see also Table 1). For example, in three studies (e.g., Bickel et al., 2016; Mellis et al., 2018a,b), the worker samples were exposed to a negative income shock (a sudden drop in financial resources, often due to job loss, illness, or relationship breakup). These workers exhibited greater delay discounting than those exposed to a financially neutral scenario (financial stability or a job change that did not affect salary or responsibilities). In other words, when faced with an abrupt economic hardship, choices prioritize immediate, low-value benefits, such as obtaining money immediately to cover urgent needs, even if this means forgoing more valuable and stable future benefits. This impulsive choice under a negative income shock, while conceptually less valuable for prioritizing small, immediate rewards over larger, delayed benefits, can also be understood as an adaptive and functional response to uncertainty and scarcity, as it allows for addressing immediate needs essential for short-term survival or stability. In this sense, Bixter and Luhmann (2013) and Bulley et al. (2016) agree that choosing the immediate reward can even be interpreted as an optimal decision in situations of economic deprivation, despite not maximizing the total value of the reward in the long run.

The findings of Bickel et al. (2016) and Mellis et al. (2018a,b) exemplify the use and effects of narratives as framing on delay discounting in work contexts. Subsequent sections will describe another series of studies that employed different framing techniques.

#### 3.3.2 Career paths and job selection

Three studies examined the relationship between delay discounting and the accumulation of human and financial capital. Saunders and Fogarty (2001) analyzed career trajectories over 36 months, focusing on choices between waiting for a higher, delayed promotion and preferring a regular, short-term promotion. The findings revealed preference reversals across the four administrations of the discounting task over time: at the first measurement, all participants preferred waiting for a better delayed promotion, whereas in the last two measurements, most participants shifted their preferences toward an immediate regular promotion. This finding is consistent with the delay discounting framework and the notion of preference reversal, in which the perceived value of a larger long-term reward decreases over time.

Hesketh (2000-ex2) examined delay discounting among novice and expert workers as a function of the waiting time for job availability and the percentage of fit with their professional career. Those choices were made under two conditions: personal decision-making and judgments about how newly graduated students would choose. The results showed that, at shorter waiting delays, experts assumed that recent graduates would discount more steeply than novices did. However, the two groups did not differ in their own choices under the personal decision condition. Nearly all workers in both groups exhibited patterns of preference reversal as waiting time increased and professional fit decreased. In other words, at short delays, participants were willing to accept a job with moderate career fit; as the time required to obtain a highly fitting job increased (i.e., larger delayed benefits), workers preferred to take an immediate job, even if it involved low professional fit.

Xu and Yin (2020) aimed to test a mediational model. The authors found that delay discounting negatively predicted delay of gratification, career commitment, job satisfaction, and overall life satisfaction. Importantly, delay of gratification and career commitment acted as mediators in the negative relationship between delay discounting and both job satisfaction and life satisfaction. In other words, workers who show greater capacity to plan and wait for future benefits, along with more substantial career commitment, tend to make more self-controlled choices, which, in turn, lead to higher perceptions of job and general life satisfaction. Delay discounting influenced job satisfaction by first affecting key self-regulatory processes. Career commitment yields both personal and organizational benefits because worker behavior is directed toward the attainment of concrete goals, even when outcomes are evaluated in the long term.

#### 3.3.3 Availability and management of resources

One group of researchers was interested in examining the degree of delay discounting and the availability of monetary resources for personal or organizational purposes. Overall, delay discounting seems to have been influenced by the amount of early retirement benefits, the perceived degree of power, the type of work, whether the resources were for personal or organizational use, and the temporary availability of those resources.

Bidewell et al. (2006) showed that middle-aged workers (*M* = 52.68, *SD* = 4.82) were willing to forgo progressively larger amounts of money to retire earlier, provided that the time to retirement decreased and that both the expected health and enjoyment during retirement increased. These findings also indirectly illuminate the phenomenon of preference reversal, insofar as the shorter the time until the preferred retirement age, the greater the amount of money (i.e., the degree of delay discounting) that a worker was willing to sacrifice to retire early.

Two studies examined the influence of power on delay discounting. In organizational contexts, power is linked to job position and responsibilities: individuals in executive roles typically have more power than those at lower hierarchical levels. Power is defined as the capacity to influence others, allocate resources, or administer sanctions (Keltner et al., 2003). In the first study, Duan et al. (2017-ex2) found that workers with a higher self-reported sense of power exhibited lower delay discounting rates (i.e., greater patience). Importantly, power alone did not fully explain differences in delay discounting; its effect varied as a function of professional frustration. Specifically, workers with high power showed lower delay discounting only when they were not frustrated. The correlation between delay discounting and sense of power was negative and weak (*r* = −0.14), whereas the association between delay discounting and optimism was moderate (*r* = −0.41). In the second study, (Joshi and Fast 2013-ex1) experimentally manipulated power by assigning workers to either a low-power condition (following instructions) or a high-power condition (assigning arithmetic tasks to another group). Consistent with the previous findings, workers in the high-power condition displayed lower delay discounting rates than those in the low-power condition.

The following studies examined the degree of delay discounting in groups of workers (Dixon et al., 2018; Hernández-Toledano et al., 2025; Logue and Anderson, 2001). For example, the findings of Dixon et al. (2018) suggest that the type of work influences intertemporal choices: waitresses in provocative clothing and exotic dancers discounted delayed monetary rewards more than waitresses who did not wear provocative clothing. The authors suggested that the work dynamics of each group could explain these findings. For example, the income of waitresses in provocative clothing or exotic dancers is often characterized by variable earnings, high dependence on tips, and a lack of stable contracts or benefits. The flow of these workers' income is present-oriented with less control, which reinforces preferences for immediate outcomes over long-term outcomes (health, career path).

The studies by Logue and Anderson (2001) and Hernández-Toledano et al. (2025) examined the delay discounting of organizational economic resources. Logue and Anderson (2001) investigated intertemporal choices in three groups: provosts, experienced fellows, and new fellows. The results showed that provosts and experienced fellows discounted delayed rewards more (i.e., showed a stronger preference for having fewer immediate resources) than new fellows, who exhibited a greater preference for larger long-term resources. Logue and Anderson (2001) suggested that, in work environments, senior administrators may experience institutional uncertainty regarding resource acquisition and have limited time horizons for spending those resources; as a result, they may prefer smaller immediate amounts that allow them to solve pressing problems or secure organizational benefits.

Logue and Anderson (2001) also hypothesized that choices might differ depending on whether the benefits are for the organization or for personal gain. Hernández-Toledano et al. (2025) addressed this question by presenting two groups of workers (managers and operative workers) with two delay discounting tasks: one focused on obtaining monetary resources for the organization and the other on obtaining personal resources. The results revealed that workers discounted organizational resources more than personal ones (fewer immediate resources for work and more delayed resources for personal use). Unlike the Logue and Anderson (2001) study, job type in the Hernández-Toledano et al. (2025) study did not influence the degree of delay discounting. Hernández-Toledano et al. (2025) also suggested that the differences in choices between organizational and personal contexts may be due to the uncertainty experienced in certain sectors regarding the acquisition and management of economic resources.

In summary, there is limited evidence that choices in discounting tasks are influenced by the type of work itself, as two of the studies identified here suggest differences according to the type of work (Dixon et al., 2018) or by type of position in the organization (Logue and Anderson, 2001), while one study revealed no differences in impulsive choice between the type of position (Hernández-Toledano et al., 2025).

In another line of study, Lahav et al. (2011) and Shavit et al. (2013) assessed the annual subjective discount rate (Equation 4) to examine the delay preferences of Israeli soldiers on active duty. The violent environment to which they are exposed, together with the high uncertainty about the future—stemming from the risk of injury, mortality, or sudden changes in deployment—reinforces the perception that time is limited and, consequently, promotes more present-oriented decisions. In both studies, delay discounting tasks in an adjusted matching format were used within the domain of labor income. Participants were asked to indicate the minimum amount they would be willing to receive at a future time *t* to forgo receiving *X* today. The participant determined the future amount. The annual subjective discount rate used to compute a delayed payment is calculated as:


$$\begin{array}{l} r=\left(\frac{P}{X}-1\right)\times \;\frac{12}{t} \end{array}$$ (4)

where *P* is the amount, the individual is willing to accept at time *t* (in months) instead of receiving an amount *X* today. The results reported by Shavit et al. (2013) showed that soldiers discounted more (i.e., exhibited lower patience) on days when the workweek ended and the weekend began, compared with the first working day of the week. Moreover, delay discounting decreased as optimism increased; that is, when soldiers felt more optimistic about the future, they tended to delay payment to receive a larger share of their salary. The studies by Lahav et al. (2011) further revealed that soldiers discounted more than both university students and adolescents.

#### 3.3.4 Inter-temporal choice and risk aversion

Only Weißmüller (2022) examined intertemporal choice and risk aversion among a sample of public- and private-sector workers. Weißmüller (2022) compared the effect of employment sector (public or private) on the degree of delay and probability discounting across three magnitudes, with participants randomly assigned to one of two framing conditions (public or private). In the public sector condition, participants were asked to imagine they worked for a public service agency. In contrast, in the private-sector condition, they were asked to imagine they worked for a for-profit company. The remaining scenario elements remained constant in both conditions: decisions involved choosing between independent investment alternatives, salary did not depend on the decisions made, long-term job security existed, and supervisors and colleagues fully trusted the worker's judgment.

The results showed that public sector workers discounted the three magnitudes by a greater amount than private sector workers did using probability discounting. In other words, public sector employees were more conservative, preferring monetary rewards that were certain to be received, regardless of their size. Private-sector workers took greater risks in their choices, opting for higher-value monetary rewards. Furthermore, public sector employees showed greater self-control (less delay discounting) for the smaller reward, whereas private sector employees were more impulsive. Another important finding was that probability discounting rates clustered under a single factor, while delay discounting rates clustered under a separate factor. Weißmüller (2022) suggests that these differences could be attributed to workers' prior sectoral experience, incentive systems, and sector-specific organizational socialization.

#### 3.3.5 Workers and health-related choices

The final category concerns delay discounting in worker samples with health-related problems, such as obesity (Mellis et al., 2018a) and tobacco use (Mellis et al., 2018b). The results of both studies showed that workers with these conditions discounted more steeply under a negative income shock framing than under a neutral one. The findings of Mellis et al. (2018a) extended to cross-commodity conditions, in which workers with obesity preferred either smaller immediate amounts of money or smaller immediate quantities of fast food rather than larger delayed amounts of either reward. Mellis et al. (2018a) suggested that high rates of delay discounting may be indirectly associated with relapse in tobacco use, as resource scarcity promotes choices focused on immediate benefits as a low-cost, fast-acting coping strategy, thereby reducing planning and the selection of options with greater long-term benefits (e.g., health). Mellis et al. (2018b) advanced a similar argument, noting that limited time and money may bias choices toward inexpensive, less healthy, and more readily accessible foods, rather than nutritionally higher-quality foods that typically cost more and require more preparation time.

Finally, only the study by Losina et al. (2017) implemented an intervention to reduce delay discounting among hospital workers. The Brigham and Women's Wellness Initiative (B-Well) consisted of a 26-week program (2 weeks of baseline and 24 weeks of intervention) designed to increase physical activity in sedentary hospital employees through financial incentives. The results showed that lower baseline delay discounting rates (greater self-control) predicted higher retention in the intervention and increased physical activity over the 6-month intervention period. This finding confirms not only that delay discounting is a state-dependent variable, but also that it is a valid behavioral indicator of change following an intervention.

## 4 Discussion

In this systematic review, we synthesized the empirical evidence that has applied the delay discounting framework to samples of workers from diverse occupational contexts. Using a robust search strategy using PRISMA guidelines, we identified 16 studies that met sufficient methodological quality criteria for inclusion in the qualitative analysis. Based on these studies, we constructed analytical categories to examine the main methodological characteristics of the delay discounting tasks employed. In addition, the findings were organized around central topics in delay discounting and organizationally relevant variables. This section discusses an integrated interpretation of the results, along with methodological considerations and recommendations derived from the available evidence, considering the literature on decision making and workplace contexts.

### 4.1 Theoretical implications for organizations

Employee behavior, including decision-making, is a key process through which organizations achieve their goals. According to normative theories (von Neumann and Morgenstern, 1947), people's choices tend to be stable, unaffected by different contexts, and oriented toward maximizing strategies to obtain the greatest long-term benefit. However, evidence from behavioral economics has shown that individuals do not always choose the greatest long-term reward; rather, depending on the magnitude of the outcome and the timing of the decision, preference reversals occur (e.g., Green et al., 1994; Green and Myerson, 2004).

In this systematic review, we found evidence of preference reversals among workers in different domains: preference for a delayed higher-quality promotion at the first assessment shifted toward an immediate regular promotion in subsequent assessments (Saunders and Fogarty, 2001); increased delays led workers to prefer immediate low-fit jobs over delayed high-fit jobs (Hesketh, 2000-ex2); and workers increasingly sacrificed money for earlier retirement, particularly when retirement was expected to be healthier and more enjoyable (Bidewell et al., 2006). The findings by Saunders and Fogarty (2001) and (Hesketh 2000-ex2) align with observational studies, such as that by Abraham et al. (2019), which reported that, after controlling for work history, previous salaries, accumulated experience, and prior advantages, the longer a person remained unemployed, the lower their likelihood of subsequently finding employment. That suggests that prolonged unemployment can reflect prior disadvantages and create new disadvantages in the future. For example, if remaining unemployed for a longer period reduces future opportunities, people will tend to accept immediate offers, even if they are worse for job performance. In this situation, accepting immediate employment with a poor professional fit can be an adaptive choice, as suggested earlier by Bixter and Luhmann (2013) and Bulley et al. (2016). The finding by Bidewell et al. (2006) is inversely related to the observational study by Mitchell and Phillips (2000), who found that if early retirement becomes less attractive (due to illness or reduced access to social security), most workers would prefer to continue working until they reach the standard retirement age. In short, the preference reversal observed in delay discounting demonstrates that preferences depend on the decision-making context rather than on individual variables.

Another common normative assumption in organizations is that a higher hierarchical position, a higher salary, or greater job tenure should be associated with greater self-control. It is often assumed that job position defines worker behavior—for example, that managers or Chief Executive Officers (CEOs) are more self-controlled, more willing to tolerate delayed outcomes, or less prone to risk-taking. However, Mumford et al. (2000a,b) argues that the skills required by different positions vary according to the type of problems to be solved, meaning that skills serve an adaptive function within organizations, and indicating that job tenure alone does not guarantee changes in decision-making.

In this systematic review, we found mixed evidence regarding the influence of work experience and job type on the degree of delay discounting. In the first place, Logue and Anderson (2001) and Hesketh (2000) support the idea that job experience affects delay discounting differently when decisions involve organizational resources or judgments about what others would choose, respectively. For example, experience and organizational seniority were associated with stronger preferences for immediate outcomes. In contrast, the findings reported by Hernández-Toledano et al. (2025) indicate that job experience does not differentiate how workers discount personal vs. organizational resources. Taken together, these findings support the notion that delay discounting is not an organizational or personal competence but rather a pattern of choice that emerges in response to specific decision contexts and contingencies. Second, the study by Dixon et al. (2018) suggested differences in delay discounting based on job type; however, the explanation given by the authors about the context of salary uncertainty faced by exotic dancers is greater than waitresses without provocative clothing was not measured as such, so the conclusion should be taken with caution.

In addition, other experimental setups may reveal differences in choices across job types or sectors. For example, in another line of research, the study by Hill et al. (2019) showed that individuals in high-risk occupations (security or health) tended to be more conservative regarding gains (choosing less risk), while workers in administrative/financial occupations showed a greater tendency toward riskier decisions in certain contexts. That relates to the study by Weißmüller (2022) identified in this review, which found that public sector workers exhibited a higher probability discounting than private sector workers. It may be that public sector workers are exposed to limited financial resources, both in quantity and time, leading them to prioritize immediate gains and lower-value benefits in their choices regarding monetary rewards. In other words, the type of daily work and experience in those contexts seems to shape how risks and rewards are assessed and how decision-making differs (Mumford et al., 2000a,b).

That leads us to consider the differences and similarities between intertemporal choice and risky choice. One way to study risky choice within behavioral economics is through the probability discounting paradigm (Rachlin et al., 1991). Several of the studies included in this review (Dixon et al., 2018; Hernández-Toledano et al., 2025; Lahav et al., 2011; Logue and Anderson, 2001; Shavit et al., 2013) invoked resource uncertainty as a complementary explanation for steeper delay discounting, proposing that when workers perceive future resources as unstable, unpredictable, or time-limited, the subjective value of delayed outcomes decreases, thereby favoring choices with immediate outcomes as an adaptive response to the risk of scarcity. However, only Weißmüller's (2022) study directly compared delay and probability discounting rates within the same group of workers. The finding that each set of discounting rates clustered into independent factors reinforces the notion that choices differ when framed in terms of delay vs. risk (Green and Myerson, 2013).

The limited evidence on probability discounting in worker samples also opens opportunities for future research to examine the extent to which organizational contexts shape risk tolerance or risk aversion, as well as whether such choice patterns depend on the type of organizational outcome. For example, workers may be more willing to assume risk when an investment promises high returns, but more risk-averse when decisions involve modifying workplace safety protocols, where increased risk could lead to accidents. Therefore, we assume that some workers might reveal differences in their choices depending on the type of work (understood as the set of activities to be performed or the experience required) and whether the alternatives differ in terms of delays, probabilities, or concurrent costs.

Mumford et al.'s (2000a,b) view about the skills as adaptive to the context and to the type of workplace problem can be linked to the previously mentioned classification proposed by (Kozioł-Nadolna and Beyer 2021), as the type of decision (strategic, tactical, or operational) depends on the worker's role, skill set, the problem at hand, the available decision alternatives, and the temporal horizon over which decision outcomes are expected to occur. Future studies could examine the effects of different time intervals (short-, medium-, and long-term) on the degree of delay discounting for distinct types of organizational outcomes (e.g., productivity, sales). Under this logic, we consider that subjective discounting valuations will vary as a function of both the outcome and the time frame over which it is realized. A sales manager (strategic decision), a team supervisor (tactical decision), and a frontline salesperson (operational decision) face choices with different temporal horizons and types of outcomes. For example, the manager may decide whether to invest today in a new information system that will reduce errors and increase productivity in 1 year; the supervisor may decide whether to implement an incentive program that could improve sales within 3 months; and the salesperson may decide whether to devote additional time today to training, which could increase commissions in the coming weeks.

Another finding worth discussing is the scarce evidence comparing mathematical models to determine the best descriptors of workers' choice behavior. The winning models in the studies that conducted such comparisons (Dixon et al., 2018; Hesketh, 2000-ex2) support prior evidence indicating that the two-parameter hyperboloid model provides a better fit to discounting data than the hyperbolic model (Green and Myerson, 2004; Myerson and Green, 1995). At the same time, evidence that the hyperbolic model outperforms the exponential model is also consistent with the phenomenon of preference reversals, or so-called irrational choice in humans (Green and Myerson, 2004).

In line with these considerations, the multidimensional model proposed by Reynolds and Schiffbauer (2004) is coherent not only at the organizational level but also with respect to discounting procedures and the empirical evidence reviewed. This approach encourages integrating other choice processes—such as probability, social, and effort discounting—to examine their combined effects on decision making. For example, Vanderveldt et al. (2015) showed that the interaction between delay and probability discounting is best described by a multiplicative model, indicating that delays and probabilities mutually influence one another. Although the studies by Bidewell et al. (2006) and (Hesketh 2000-ex2) combined delays and percentages within the same tasks, their results do not allow conclusions about whether additive or multiplicative discounting models better explain workers' choices. Future research could therefore explore multidimensional discounting models in worker populations to determine which combinations of costs differentially affect discounting rates.

### 4.2 Methodological recommendations

Based on the studies analyzed, we identified some methodological recommendations for researchers seeking to expand knowledge of the variables that influence delay discounting in workplace contexts. The first recommendation concerns study samples. For example, the articles by Hesketh et al. (1998), as well as six additional studies by Duan et al. (2017), Hesketh (2000), and Joshi and Fast (2013), could not be included in this review because they relied on samples of university students. This limitation has been acknowledged by the authors themselves, as even when the narratives or choice-task content are framed in workplace contexts, students' lack of work experience reduces the ecological validity of the resulting data. Therefore, we recommend using worker samples for these types of studies whenever possible to increase the ecological validity of the findings. For example, studies on impulsive choices associated with substance use necessarily require that the tasks be administered to groups of users, so that differences in the degree of discounting can be observed (Mejía-Cruz et al., 2016).

A second recommendation for future studies is to provide more detailed descriptions of organizational variables that allow for a richer characterization of worker samples. Only a minority of the included studies (Hernández-Toledano et al., 2025; Weißmüller, 2022) reported a broader set of job-related variables, such as years of experience in the position, sector type, monthly salary, types of activities performed, supervisory responsibilities, and whether participants managed organizational resources and of what kind. Reporting such information would enhance the explanatory, interpretive, and applied value of findings by enabling stronger hypotheses regarding the influence of demographic and organizational variables on impulsive and risky choice, thereby supporting contextual or environmental explanations rather than trait-based accounts of worker behavior. Detailed participant descriptions would also increase the external validity of the findings by facilitating comparisons across sectors, hierarchical levels, and organizational contexts.

Another trend observed in this review is that five of the included studies used the MTurk platform for data collection. MTurk provides paid crowdsourcing work to individuals who complete surveys on a full- or part-time basis, typically without a formal employment contract. Only Bickel et al. (2016) explicitly noted that, although participants came from a shared labor pool (MTurk), they differed in terms of work schedules, and some even identified as unemployed or as students. This highlights the heterogeneity of MTurk samples and further underscores the need for detailed reporting of organizational and demographic variables. Limited-resource scenarios have been shown to promote impulsive choice (Bickel et al., 2016; Mellis et al., 2018a,b). Consequently, MTurk workers with multiple sources of income may exhibit discounting rates different from those who rely on MTurk as their sole source of income, as greater financial stability is likely to buffer against scarcity-driven, present-oriented decision-making. Shimoni and Axelrod (2025) further argue that when researchers rely on data collected via MTurk, they should preferentially recruit Master Workers, who are less prone to attentional errors in online tasks. Among the studies included in this review, only Bickel et al. (2016) reported that participants had passed a Human Intelligence Task (HIT) qualification. Accordingly, we recommend that future studies using MTurk as a data source ensure that participants pass attention or quality-control filters appropriate for online experimental tasks and provide a more detailed characterization of participants' demographic and occupational variables.

Another methodological issue concerns studies that examined delay discounting in workers with health-related conditions, such as obesity (Mellis et al., 2018a) and tobacco use (Mellis et al., 2018b). These studies did not include comparison groups, making it difficult to determine whether the observed participants would discount future outcomes more steeply under adverse financial scenarios than workers without such health conditions. The absence of comparison groups limits internal validity, as heightened impulsivity cannot be confidently attributed to the health condition itself; other factors, such as job-related stress or income level, may also account for the findings. Additional implications include potential selection bias and restricted generalizability to other worker populations. Therefore, we recommend that future research on intertemporal choice among workers with health-related conditions incorporate appropriate comparison groups to strengthen inferences about health-related decision-making and enhance the external validity of the findings.

In addition to the last point, it is worth noting that discounting measures are commonly used in clinical and translational research as behavioral markers of impulsivity, self-control, and sensitivity to reinforcement and aversive events (Amlung et al., 2017; Bickel et al., 2012). For example, in the field of addiction, impulsivity is associated with sensitivity to and preference for the apparent immediate rewards of substance use (euphoria, relaxation), at the expense of sensitivity to delayed consequences (harmful health effects, job loss, family problems). However, sensitivity to delays in obtaining an outcome or to the magnitude of the results exists on a continuum of individual variation. That is why the phenomenon of delay discounting is observed across different study groups and in most of the individuals evaluated. In other words, people discount to some extent, but certain historical conditions, present contexts, or possible future situations allow us to observe differences between groups of clinical and social interest. Therefore, we maintain that using delay discounting as a behavioral marker of choice should not be understood to study *abnormal* behavior.

Another important aspect to consider, which limits the direct comparison of the findings reported by the studies included in this review, is the heterogeneity in the types of outcomes used. The meta-analysis by Odum et al. (2020) demonstrated that, with few exceptions, non-monetary outcomes (food, drink, health, drugs, etc.) were discounted more than monetary outcomes across a variety of procedures and subsamples, a finding that reflects a characteristic of delay discounting as a state variable. Odum et al. (2020) proposed the Decreasing Future Preference Hypothesis to explain these findings, based on the premise that many non-monetary outcomes lose relevance over time because our tastes, needs, or priorities change; that is, these outcomes are tied to present contexts or needs. In contrast, money maintains its subjective value better because it is flexible or fungible: we can exchange it for other goods or services in the future. For example, a slice of pizza in the future might lose more value (be discounted more) because you might not want to eat it later. However, having $50 in the future retains its value better because people can exchange it for any other good of similar value.

Regarding the studies in this review that used non-monetary outcomes such as air quality, job fit, and career advancement, we might expect that these would be discounted more than monetary outcomes, given their relevance and need in a specific context and their high value in the present, both at the organizational and individual worker levels. However, without a direct comparison with monetary outcomes, testing the Decreasing Future Preference Hypothesis is not plausible with the available evidence from workers. Future research would benefit from greater standardization of delay discounting procedures and from direct comparisons of monetary and job outcomes within the same experimental designs.

The discounting literature has also shown evidence that the time horizon of the delays and the magnitudes used in human studies have distinct effects on the degree of delay discounting. Mathematical models such as the hyperbolic and hyperboloid models maintain that the subjective value of outcomes decreases non-linearly as delays increase; that is, shorter delays tend to decrease subjective value more than longer delays (Green and Myerson, 2004). Furthermore, experimental research has consistently documented the magnitude effect, where lower-value rewards are discounted more than higher-value rewards (e.g., Green et al., 2014).

The studies selected for this review differed considerably in both the number of delays and the length of the intervals, ranging from short delays (e.g., hours or days) to extended time horizons of several years (25 years). This methodological heterogeneity is relevant because delay-discounting estimates can vary across time ranges assessed, delay distributions, and reward magnitudes (Mitchell and Wilson, 2010). For example, tasks focused on immediate outcomes (e.g., food, drink, drugs) may capture impulsive processes or a preference for immediate gratification. In contrast, tasks with long time horizons might involve more abstract, prospective processes (e.g., retirement savings). Besides, almost half of the included studies used different procedures to calculate indifference points. The evidence suggests that there are no robust changes in delay discounting across the titrating and fixed sequence procedures (e.g., Rodzon et al., 2011). However, other evidence indicates changes in the presentation format—e.g., the order of delays and the descending-ascending order (e.g., Robles and Vargas, 2007). Overall, direct comparisons between the included studies should be interpreted with caution.

Regarding data analysis, because most of the studies included in this review relied on singular framings or narratives, the use of the systematicity criteria proposed by Johnson and Bickel (2008), as well as the assessment of goodness of fit of mathematical models, becomes a methodological need to establish the internal validity of the experimental tasks. Accordingly, we recommend that future studies examining variables or contexts that influence delay discounting in worker samples incorporate these indicators to strengthen conclusions about the internal validity of the data. This recommendation also has theoretical implications, as it would allow researchers to evaluate the extent to which different mathematical models of discounting (e.g., hyperbolic, hyperboloid, exponential) are equally adequate not only in organizational contexts, but also across the specific variables that affect or modulate discounting rates. For instance, although studies by Saunders and Fogarty (2001), Hesketh (2000), and Bidewell et al. (2006) reported evidence suggestive of preference reversals, there is insufficient information regarding the goodness of fit of the mathematical models used.

A second implication of the prior recommendation is that it would enable the identification of subgroups of choice patterns within worker samples, which may otherwise be obscured by conclusions based on aggregated data that mask choice dynamics associated with organizational contexts (e.g., Bickel et al., 2016; Myerson et al., 2001). A third implication concerns external validity, as the use of systematicity and model-fit indicators would reduce ambiguities regarding the comparability of data obtained from workers with those from other populations (e.g., individuals with substance use disorders or pathological gambling), and would also facilitate future meta-analyses aimed at quantifying the effects of methodological variations or interventions on temporal discounting rates.

In addition, when two or more mathematical models are tested, we extend the recommendation of Burnham and Anderson (2004) to use robust model-comparison indices, such as the mean square error, the Akaike Information Criterion (AIC), the second-order Akaike Information Criterion (AICc), or the Bayesian Information Criterion (BIC). Finally, the limited evidence from individual-level analyses in the reviewed studies highlights the need to incorporate them, not only because they are central to the behavioral analysis of choice, but also because, within organizational settings, one of the aims of research is often to address individual-level problems. As is well known, aggregated data can obscure individual performance on discounting tasks, a limitation that is even greater when the goal is to design or implement organizational interventions.

### 4.3 Applied implications for organizations

In organizational settings, the goal of a psychologist or behavior analyst is to address issues related to individual employee behavior and its interaction with the work environment (López, 2008). As discussed in the previous section, recommendations to provide more detailed descriptions of workers' demographic and organizational variables, as well as to include indicators of the data's internal validity, would facilitate the design of more precise interventions tailored to employees‘ characteristics and their organizational context.

Although intervention is a central focus in applied research within organizations, only one study included in this review (Losina et al., 2017) implemented an intervention to increase physical activity among sedentary employees, using delay discounting as a behavioral marker of a change from impulsive to more self-controlled choices. Delay discounting had two key implications in this study: first, it reliably predicted participants' adherence to the intervention; and second, it was negatively associated with the number of steps taken per week. In other words, lower impulsivity was linked to greater physical activity and higher intervention adherence. These findings replicate and extend the results reported by Sofis et al. (2016), who observed decreases in delay discounting as a physical activity intervention progressed, and are inversely related to the findings of Campbell et al. (2021), who reported that higher delay discounting was associated with lower treatment adherence in patients with diabetes. Collectively, these results align with the meta-analysis by Rung and Madden (2018), which identified delay discounting as a variable sensitive to interventions or self-control training.

Therefore, although to date only one study has demonstrated behavioral change alongside reduced delay discounting rates following an intervention in workers, evidence from other studies suggests that delay discounting is a viable and valid metric for use in organizational settings when implementing interventions. Translational research in occupational health and safety could improve understanding of limiting and facilitating factors at the individual, social, and organizational levels, increasing the likelihood that research outcomes lead to tangible improvements in employee wellbeing through effective interventions.

While delay discounting is not an intervention technique, it is a valid behavioral indicator supported by a broad evidence base, highlighting its relevance as a decision-making process linked to a lack of self-control. This ability is crucial not only in the workplace but also in daily life. From this perspective, applying the delay discounting paradigm could aid in identifying and describing the relationship between impulsive choice and various organizational factors, such as productivity, time management, and individual health and safety strategies, providing a reliable indicator of behavioral change during and after interventions or training programs.

A final consideration regarding the intervention implemented by Losina et al. (2017) is that it relied on financial incentives, which may not always be feasible in other countries or organizational contexts. Limited feasibility could stem from high costs, such as purchasing technological devices for all participants, especially in studies conducted with constrained resources. Furthermore, promoting physical activity among employees—or participants more broadly—should ideally be supported by skill development rather than monetary rewards, enhancing the ecological validity of the interventions. The systematic review by Ramezani et al. (2022) identified several effective strategies for increasing workers' physical activity: motivation and support, monitoring and feedback, and information and education.

### 4.4 Evidence for external validity

We identified representative findings in the field of delay discounting that have been replicated across some of the 16 studies included in this systematic review. For example, Hernández-Toledano et al. (2025), Lahav et al. (2011), Shavit et al. (2013), and Weißmüller (2022) observed a magnitude effect, in which small-to-medium rewards were discounted more steeply than larger, delayed rewards. A similar magnitude effect was also observed in probability discounting, but in a transitive and reverse order (*k*_small < *k*_medium < *k*_large), indicating that smaller rewards were associated with riskier choices than medium or large rewards. Weißmüller's (2022) findings suggest that workers demonstrate greater willingness to wait and increased risk aversion when larger monetary rewards are at stake.

Bickel et al. (2016) replicated the sign effect, in which gains are discounted more steeply than monetary losses, in both past- and future-oriented choices. In other words, when outcomes are framed as future gains, workers are less willing to wait for larger, delayed benefits (“better to gain something now”). In contrast, when outcomes are framed as losses or future payments, workers prefer to postpone the loss or payment (“better to lose something later”). This finding aligns with prospect theory and loss aversion (Loewenstein, 1987; Tversky and Kahneman, 1981).

### 4.5 Limitations of the current review

Although we used several terms synonymously in the two article search strings, we were unaware that the online paid collaboration platform Amazon Mechanical Turk (MTurk) is a growing network of workers increasingly used for data collection in the social sciences and behavior analysis (Shimoni and Axelrod, 2025). As we progressed through the article review, we observed the platform's use as a data source. Consequently, terms such as “Mechanical Turk” or “MTurk” were not included in the search of titles, abstracts, and keywords, which may have limited the number of articles identified.

## 5 Conclusions

This systematic review demonstrates that the delay discounting paradigm is a valuable tool for understanding impulsive decision-making in workplace contexts, highlighting how individual factors (optimism, sense of power, health status), contextual factors (job type, available resources, work sector), and methodological factors (framing, reward magnitude, task type) influence discounting rates. The findings show consistent effects, including preference reversal, magnitude and sign effects, and distinctions between delay and probability discounting. These findings were consistent with Mumford et al.'s perspective (2000a; 2000b) and with a basic behavioral principle: individuals discriminate among contingencies (Skinner, 1953).

In addition, a contextual and scientific approach to worker behavior would be more informative and better suited to designing interventions, as it aligns with core behavioral principles: behavior is modifiable and current behavior is the product of learning histories and prior exposure to different contingency structures (Skinner, 1953, 1969). Explaining the influence of the environment on worker behavior thus clarifies that such behavior is adaptive and context-dependent, rather than the expression of a fixed trait.

The review also identifies methodological limitations, including limited characterization of organizational variables and the need to ensure data internal validity through mathematical models and systematicity criteria. Overall, the evidence suggests that delay discounting is a sensitive and applied marker for assessing self-control and designing interventions in organizational settings, providing a framework for linking impulsive choices to productivity, wellbeing, and resource management strategies at work.

## Data Availability

The original contributions presented in the study are publicly available. This data can be found here: https://osf.io/p6a5r?view_only=4e29f6e68c9e4c00a6bbb7a61285e248.
