# Supplementary material for: A systematic review of delay discounting among workers: framing effects, resource availability, and health
Source: Front Psychol. 2026 Jul 13;17:1801136. doi: 10.3389/fpsyg.2026.1801136 (PMC13402185; doi:10.3389/fpsyg.2026.1801136)
Supplement: Supplementary file 1 [file Table_1.docx]

**Supplementary** **material**

**Table S1.** Studies were assessed using the NIH Quality Assessment Tool for Observational Cohort and Cross-sectional Studies.

| **Study** | **Was the research question or objective in this paper clearly stated?** | **Was the study population clearly specified and defined?** | **Was the participation rate of eligible persons at**  **least 50%?** | **Were all the subjects selected or recruited from the same or similar populations?** | **Was a sample size justification, power description, or variance and effect estimates provided?** | **For the analyses in this paper, were the exposure(s) of interest measured prior to the outcome(s) being measured?** | **Was the timeframe sufficient so that one could reasonably expect to see an association between exposure and outcome if it existed?** | **For exposures that can vary in amount or level, did the study examine different levels of the exposure?** | **Were the exposure measures (independent variables) clearly defined, valid, reliable, and implemented consistently across all study participants?** | **Was the exposure(s) assessed more than once over time?** | **Were the outcome measures (dependent variables) clearly defined, valid, reliable, and implemented? consistently across all study participants?** | **Were the outcome assessors blinded to the exposure status of participants?** | **Was loss to follow-up after baseline 20% or less?** | **Were key potential confounding variables measured and adjusted statistically for their impact on the relationship? between exposure(s) and outcome(s)?** | **Total** | **Summary Quality** |
| --- | --- | --- | --- | --- | --- | --- | --- | --- | --- | --- | --- | --- | --- | --- | --- | --- |
| Bickel et al. (2016) | **✓** | **✓** | **NA** | **✓** | **✓** | **NA** | **NA** | **✓** | **✓** | **NA** | **✓** | **NA** | **NA** | **✓** | **8** | **i** |
| Bidewell et al., 2006 | **✓** | **✓** | **NA** | **✓** | **NR** | **NA** | **NA** | **✓** | **✓** | **NA** | **✓** | **NA** | **NA** | **✓** | **7** | **i** |
| Dixon et al., 2018 | **✓** | **✓** | **NA** | **✓** | **NR** | **NA** | **NA** | **✓** | **✓** | **NA** | **✓** | **NA** | **NA** | **✓** | **7** | **i** |
| Duan et al., 2017-ex2 | **✓** | **✓** | **NA** | **✓** | **NR** | **NA** | **NA** | **✓** | **✓** | **NA** | **✓** | **NA** | **NA** | **NA** | **6** | **i** |
| Hernández et al., 2025 | **✓** | **✓** | **NA** | **✓** | **NR** | **NA** | **NA** | **✓** | **✓** | **✓** | **✓** | **NA** | **NA** | **NA** | **7** | **i** |
| Hesketh 2000-ex2 | **✓** | **✓** | **NA** | **✓** | **X** | **NA** | **NA** | **✓** | **✓** | **NA** | **✓** | **NA** | **NA** | **✓** | **7** | **i** |
| Joshi & Fast, 2013-ex1 | **✓** | **✓** | **NA** | **✓** | **✓** | **NA** | **NA** | **✓** | **✓** | **NA** | **✓** | **NA** | **NA** | **✓** | **8** | **i** |
| Lahav et al., 2011 | **✓** | **✓** | **NA** | **✓** | **✓** | **✓** | **NA** | **✓** | **✓** | **NA** | **✓** | **NA** | **NA** | **✓** | **9** | **i** |
| Logue & Anderson, 2001 | **✓** | **✓** | **NA** | **✓** | **NR** | **NA** | **NA** | **✓** | **✓** | **✓** | **✓** | **NA** | **NA** | **NA** | **7** | **i** |
| Mellis et al., 2018a | **✓** | **✓** | **NA** | **✓** | **🗶** | **NA** | **NA** | **✓** | **✓** | **NA** | **✓** | **NA** | **NA** | **✓** | **7** | **i** |
| Mellis et al., 2018b | **✓** | **✓** | **NA** | **✓** | **🗶** | **NA** | **NA** | **✓** | **✓** | **NA** | **✓** | **NA** | **NA** | **✓** | **7** | **i** |
| Saunders & Fogarty, 2001 | **✓** | **✓** | **NA** | **✓** | **🗶** | **NA** | **NA** | **✓** | **✓** | **NA** | **✓** | **NA** | **NA** | **🗶** | **6** | **i** |
| Shavit et al., 2013 | **✓** | **✓** | **NA** | **✓** | **🗶** | **NA** | **NA** | **✓** | **✓** | **NA** | **✓** | **NA** | **NA** | **✓** | **7** | **i** |
| Weibmüller, 2022 | **✓** | **✓** | **NA** | **✓** | **🗶** | **NA** | **NA** | **✓** | **✓** | **NA** | **✓** | **NA** | **NA** | **✓** | **7** | **i** |
| Xu & Yin, 2020 | **✓** | **✓** | **NA** | **✓** | **✓** | **NA** | **NA** | **✓** | **✓** | **NA** | **✓** | **NA** | **NA** | **✓** | **8** | **i** |

*Note.* Quality was rated as 0 for **poor** (0–4 out of 14 questions), **i** for **fair** (5–10 out of 14 questions), or **ii** for **good** (11–14 out of 14 questions); **NA**: not applicable, **NR**: not reported.
