# Supplementary material for: A systematic review of delay discounting among workers: framing effects, resource availability, and health
Source: Front Psychol. 2026 Jul 13;17:1801136. doi: 10.3389/fpsyg.2026.1801136 (PMC13402185; doi:10.3389/fpsyg.2026.1801136)
Supplement: Supplementary file 2 [file Table_2.docx]

**Supplementary** **material**

**Table S2.** Studies were assessed using the NIH Quality Assessment Tool for Before-After (Pre-Post) Studies With No Control Group.

| **Study** | **1. Was the study question or objective clearly stated?** | **2. Were eligibility/selection criteria for the study population prespecified and clearly described?** | | **3. Were the participants in the study representative of those who would be eligible for the test/service/intervention in the general or clinical population of interest?** | **4. Were all eligible participants that met the prespecified entry criteria enrolled?** | **5. Was the sample size sufficiently large to provide confidence in the findings?** | **6. Was the test/service/intervention clearly described and delivered consistently across the study population?** | **7. Were the outcome measures prespecified, clearly defined, valid, reliable, and assessed consistently across all study participants?** | **8. Were the people assessing the outcomes blinded to the participants' exposures/interventions?** | **9. Was the loss to follow-up after baseline 20% or less? Were those lost to follow-up accounted for in the analysis?** | **10. Did the statistical methods examine changes in outcome measures from before to after the intervention? Were statistical tests done that provided p values for the pre-to-post changes?** | **11. Were outcome measures of interest taken multiple times before the intervention and multiple times after the intervention (i.e., did they use an interrupted time-series design)?** | **12. If the intervention was conducted at a group level (e.g., a whole hospital, a community, etc.) did the statistical analysis take into account the use of individual-level data to determine effects at the group level?** | **TOTAL** | **Summary Quality** |
| --- | --- | --- | --- | --- | --- | --- | --- | --- | --- | --- | --- | --- | --- | --- | --- |
| Losina et al., 2017 | ✓ | ✓ | ✓ | | ✓ | CD | ✓ | ✓ | NR | ✓ | ✓ | ✓ | ✓ | 10 | ii |

*Note.* Quality was rated as 0 for **poor** (0–3 out of 12 questions), **i** for **fair** (4–9 out of 12 questions), or **ii** for **good** (10–12 out of 12 questions); **NA**: not applicable, **NR**: not reported, **CD**: cannot determine.

NIH quality assessment tool was available at: <https://www.nhlbi.nih.gov/health-topics/study-quality-assessment-tools>
